# Pig Urinary Concentration of Mycotoxins and Metabolites Reflects Regional Differences, Mycotoxin Intake and Feed Contaminations

**DOI:** 10.3390/toxins11070378

**Published:** 2019-06-30

**Authors:** Lucia Gambacorta, Monica Olsen, Michele Solfrizzo

**Affiliations:** 1 Institute of Sciences of Food Production (ISPA), National Research Council (CNR), Via Amendola 122/O, 70126 Bari, Italy; 2 National Food Agency, Department of Risk Benefit Assessment, P.O. Box 622, 751 26 Uppsala, Sweden

**Keywords:** mycotoxins, biomarkers, urine, UPLC-MS/MS, intake, feed, grain

## Abstract

The determination of mycotoxin and metabolite concentrations in human and animal urine is currently used for risk assessment and mycotoxin intake measurement. In this study, pig urine (*n* = 195) was collected at slaughterhouses in 2012 by the Swedish National Food Agency in three counties representing East, South and West regions of Sweden. Urinary concentrations of four mycotoxins, (deoxynivalenol (DON), zearalenone (ZEA), fumonisin B_1_ (FB_1_), and ochratoxin A (OTA)), and four key metabolites, (deepoxy-deoxynivalenol (DOM-1), aflatoxin M_1_ (AFM_1_, biomarker of AFB_1_), α-zearalenol (α-ZOL), and β-zearalenol (β-ZOL)) were identified and measured by UPLC-MS/MS. Statistically significant regional differences were detected for both total DON (DON + DOM-1) and total ZEA (ZEA + α-ZOL + β-ZOL) concentrations in pig urine from the three regions. These regional differences were in good agreement with the occurrence of *Fusarium graminearum* mycotoxins (DON + ZEA) in cereal grains harvested in 2011 in Sweden. There were no statistically significant differences in FB_1_, AFM_1_ and OTA urinary concentrations in pigs from the three regions. The overall incidence of positive samples was high for total ZEA (99–100%), total DON (96–100%) and OTA (85–95%), medium for FB_1_ (30–61%) and low for AFM_1_ (0–13%) in the three regions. Urinary mycotoxin biomarker concentrations were used to estimate mycotoxin intake and the level of mycotoxins in feeds consumed by the monitored pigs. The back-calculated levels of mycotoxins in feeds were low with the exception of seven samples that were higher the European limits.

## 1. Introduction

Deoxynivalenol (DON), zearalenone (ZEA), fumonisin B_1_ (FB_1_), ochratoxin A (OTA) and aflatoxin B_1_ (AFB_1_) were defined as the five agriculturally important mycotoxins for their impact on the safety of human food and feed [1]. Pigs are quite susceptible to mycotoxin toxicity; therefore, the guidance values for cereals and cereal products and compound feed for animal feeding are lower for those destined for pigs [2]. In developed countries, feeds are constantly monitored for mycotoxins to protect animal health and increase animal productivity and farmer’s income. Monitoring mycotoxins in cereal grain is expensive and laborious due to the inhomogeneous distribution of mycotoxins in raw materials, which requires the application of adequate sampling plans that comprise the collection of a high number of samples to be analyzed. Hult et al. (1979) demonstrated the efficacy of biomonitoring of OTA in pig’s blood to determine OTA levels in feeds consumed by the monitored pigs [3]. Subsequently, Gilbert et al.; (2001) demonstrated a better correlation between OTA consumption and urinary OTA as compared to blood OTA [4]. More recently, with the availability of better performing LC-MS/MS instrumentation, new and high performing analytical methods, such as those reviewed by Vidal et al.; (2018) [5], were developed for multiple mycotoxins and their phase I and II metabolites determination in biological fluids, especially urine. The introduction of an enzymatic digestion step in the sample preparation of urine was used in several analytical methods in order to hydrolyze conjugated mycotoxins, phase II metabolites and conjugated phase I metabolites of mycotoxins into free analytes. This approach reduces the number of analytes to be monitored, increases the sensitivity of the analytical method and avoids producing/synthesizing commercially unavailable conjugated standards. A good dose response was demonstrated in piglets between the consumption of the five agriculturally important mycotoxins and urinary concentrations of mycotoxins and their metabolites [6]. Urinary concentrations of mycotoxins and their metabolites were also successfully used to demonstrate the efficacy of grape pomace to significantly reduce the bioavailability of AFB_1_ and ZEA in pigs [7]. Few studies have been conducted in the past to assess pig exposure to mycotoxins, and most of them considered only one mycotoxin and its metabolites [8,9,10,11,12,13]. The main aim of the present work was to assess pig exposure to the five agriculturally important mycotoxins through the determination of mycotoxin biomarkers in urine collected at slaughterhouses in three regions of Sweden and to evaluate any regional differences. The main conclusion of this study was that mycotoxin exposure of Swedish pigs was low with a few exceptions. Moreover, our results indicate that pig urine can be used for monitoring both pig exposure to mycotoxins as well as the trends of DON and ZEA in cereal grains. 

## 2. Results and Discussion

### 2.1. Occurrence, Intake, Feed Contamination and Regional Differences 

The UPLC-MS/MS method and MS/MS parameters used in this study to analyze pig urine were previously reported [6,7]. As a minor modification, 5 mL of urine was used instead of 6 mL. The method performance was verified by a recovery experiment conducted with Swedish pig urine. As shown in Table 1, mean recoveries (>70%) and the repeatability of results (1–12%) were all acceptable, with the exception of FB_1_ that gave a mean recovery of 64% but a good repeatability of results (7%). In Table 1, we also report the values of the limit of detection (LOD) (0.006–0.36 ng/mL) and the limit of quantification (LOQ) (0.02–1.21 ng/mL) for each analyte. LOD and LOQ were calculated as three times and 10 times the noise, respectively. 

In Table 2, we report the incidence of positive samples, and the mean, median and max concentration of each analyte, reported as ng/mL and as ng/mg creatinine (crea). The total concentrations of DON (DON + DOM-1) and ZEA (ZEA + α-ZOL + β-ZOL) are also reported. The high percentages of samples positive for DON, ZEA and their metabolites was not surprising and were comparable to those previously reported by other authors for pig urine [9,11,13].

**Figure 1:**
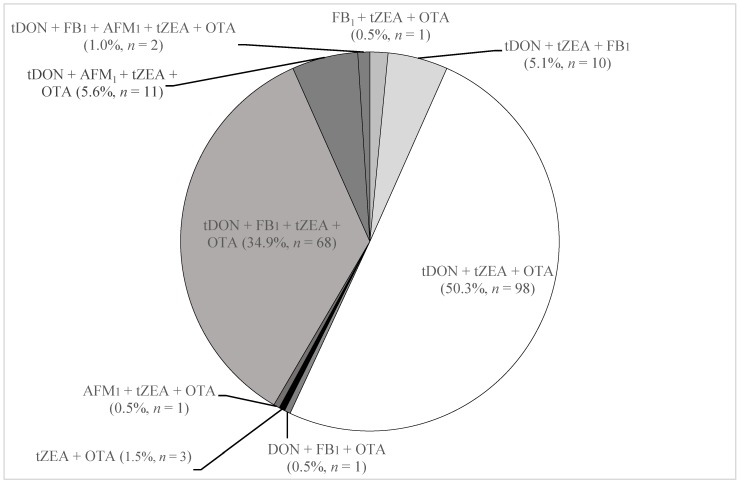
Mycotoxin combinations and their incidence in 195 Swedish pig urine samples collected in 2012. tDON = total DON; tZEA = total ZEA.

The occurrence of multiple mycotoxins and metabolites in pig urine is reported in Figure 1. The most common combinations were total DON + total ZEA + OTA (50.3% of samples) followed by total DON + FB_1_ + total ZEA + OTA (34.9% of samples) (Figure 1). Each of the seven other combinations occurred in less than 6% of urine. 

In Table 3, we compare the results of the present study (percentage of positives and mean concentrations of positive samples) with those reported in other studies conducted with pigs or piglets bred in six different countries. Mean concentrations of mycotoxin biomarkers in Swedish pigs were within the concentrations reported by other authors for this animal species. In particular, the urinary mean DON concentration of Swedish pigs was lower than that measured in Belgian pigs [12] but higher than those measured in Vietnamese pigs [13] and French piglets [7]. Mean urinary concentrations of ZEA and total ZEA were lower than those reported from Croatia [9,10], Austria [11] and Vietnam [13] but higher than those reported from France for piglets [7]. A high mean concentration of ZEA (206 ng/mL) was reported in Croatia for 30 pigs showing clear symptoms of hyperestrogenism, probably because they were fed with feed highly contaminated by ZEA [9]. Swedish pig urine showed incidences of positive samples and a mean OTA concentration higher than those reported for Belgian pigs and French piglets [7,12]. On the other hand, the incidence of positive samples and mean concentration of FB_1_ in Swedish pigs was comparable to those reported for Belgian pigs and French piglets [7,12]. A remarkable difference was observed when comparing the AFM_1_ results of Swedish and Vietnamese pigs. As reported in Table 3, the Swedish pig urine showed an incidence of positive samples and mean AFM_1_ concentration 8–11 and 4–27 times lower compared to Vietnamese pig urine, respectively [8,13]. These results confirmed that the feeds used to fed Swedish pigs were only sporadically and marginally contaminated by AFB_1_ which is mainly excreted in urine as AFM_1_. Taken together, the results reported in Table 3 gave a picture about the differing occurrence of the eight biomarkers of the five agriculturally important mycotoxins in the urine of pigs bred in Sweden and France, and for 1–6 biomarkers of 1–3 mycotoxins in five more countries. 

The urinary mycotoxin and metabolite concentrations measured in this study and the urinary excretion rate of the five target mycotoxins and their metabolites in pigs [6] were used to calculate the probable daily intake (PDI) of the target mycotoxins in Swedish pigs using Formula (1). The calculated mean and max PDI values of each mycotoxin in exposed pigs is shown in Table 4. 

The mean PDI value of DON was higher than those of ZEA, OTA, FB_1_ and AFB_1_. The calculated PDI values, mean pig weight, mean daily urine volume and mean weight of feed consumed daily by pigs were used to calculate the level of each mycotoxin in the feed consumed by each pig monitored in this study according to Formula (2), reported in the experimental section. The results of this evaluation are reported in Table 5, which also shows the maximum permitted level of AFB_1_ [14] and the guidance levels of DON, ZEA, OTA and fumonisins in feeds for pigs [2]. The estimated mean level of DON in feed was 116.8 µg/kg, whereas for ZEA, OTA, FB_1_ and AFB_1_, they ranged from 16.1–0.6 µg/kg. These values are largely below the limits for DON (900 µg/kg), ZEA (250 µg/kg), OTA (50 µg/kg), AFB_1_ (20 µg/kg) and FB_1_ (5000 µg/kg). However, the estimated mycotoxin levels of the feeds consumed by seven pigs were higher the limits of DON, OTA or AFB_1_. Five out of the seven pigs were bred in the West region and three of them consumed feed with high DON levels, whereas two pigs consumed feeds with high levels of OTA (Table 5). On the other hand, only one pig from the East region and one from the South region consumed feed contaminated with high levels of AFB_1_ and OTA, respectively. 

**Figure 2:**
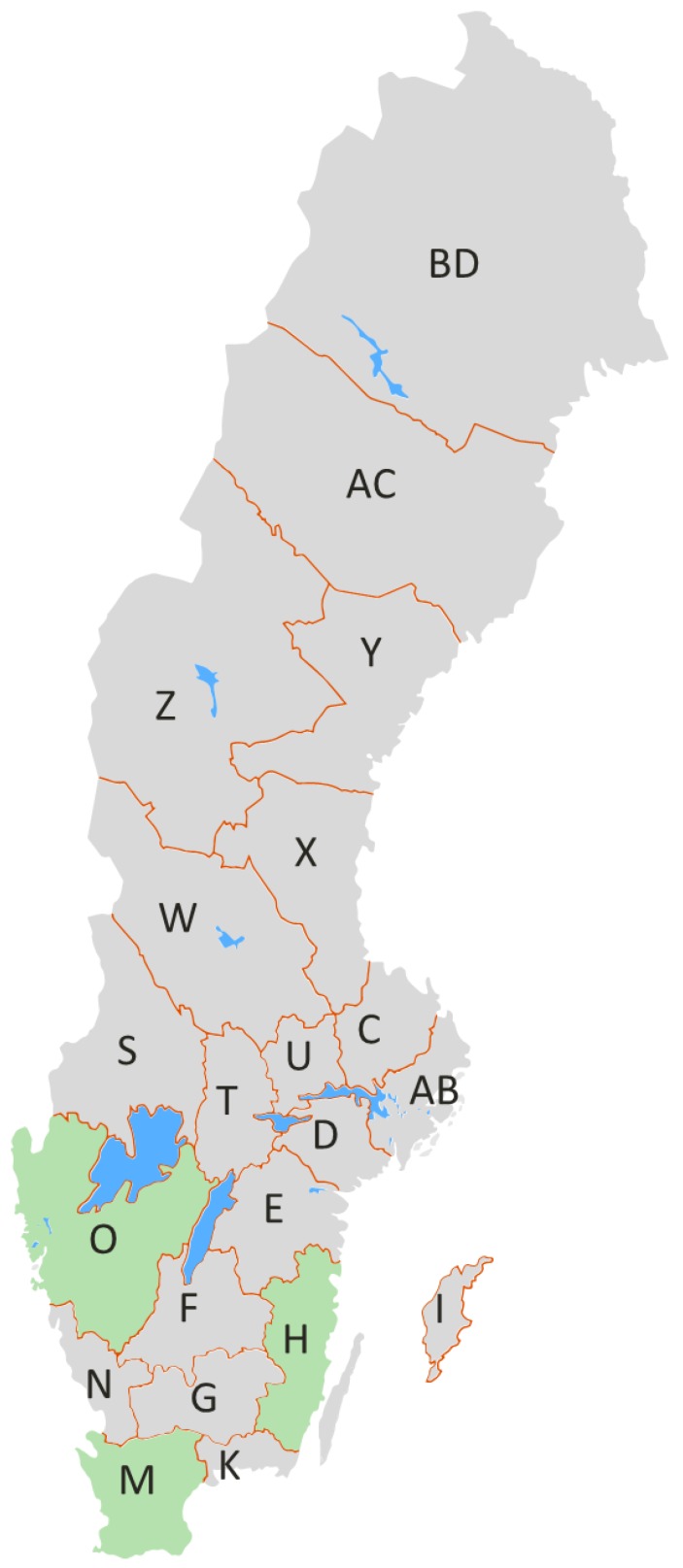
Map of the Sweden showing from which county the pig urine samples were collected. The West region is represented by Västra Götaland county (O), the South region is represented by Skåne county (M) and the East region is represented by Kalmar county (H). The names of other counties are reported here https://www.iso.org/obp/ui/#iso:code:3166:SE.

These results prompted us to statistically compare the mycotoxin concentrations in the urine of pigs bred in the West, South and East regions of Sweden (Figure 2). The results of this statistical evaluation were reported in Table 6 as ng/mL and as ng/mg crea. A significant difference (*p* < 0.001) was observed for total DON between West (56.8 ± 88.7 ng/mL) and South (19.3 ± 22.8 ng/mL) and between West and East (12.8 ± 13.6 ng/mL). The statistical analyses of crea adjusted urine concentrations confirmed the significant difference (*p* < 0.009) of total DON between West (45.7 ± 106 ng/mg crea) and South (12.9 ± 15.1 ng/mg crea), but no differences were observed between West and East and between South and East. Total ZEA was significantly higher (*p* < 0.009) in the West (7.8 ± 10.4 ng/mL) compared to South (4.3 ± 9.5 ng/mL) but no significant difference was found to the East. Similar results were obtained for crea adjusted concentrations, although a significant difference (*p* < 0.05) was also observed between East (12.5 ± 42.2 ng/mg crea) and South (3.7 ± 10.5 ng/mg crea).

These regional differences were in good agreement with the differing occurrence of *Fusarium graminearum* mycotoxins (DON and ZEA) in cereal grains harvested in 2011 in different Swedish regions [15,16]. Moreover, data pertaining to DON in oats in 2011 collected by the Swedish grain industry (Thomas Börjesson, personal communication) showed similar differences between West (mean level 4287 ± 4120 µg/kg, *n* = 1300) and South (mean level 572 ± 655 µg/kg, *n* = 160). Despite the fact that pigs are not fed oats, the data reflect the situation of a high infection rate with *F. graminearum* in the fields of the West region, which was particularly troublesome in oats this year.

According to the Kruskal–Wallis equality of population rank test, there was no difference (*p* > 0.05) in FB_1_, AFM_1_ and OTA between the three regions. Considering that AFB_1_ and FB_1_ were not found in Swedish grains, the positive samples were most likely due to other feed components or the farmer was not producing their own feed and used commercial feed; in that case, we should not expect regional differences. Protein concentrates were found to contain AFB_1_ from 5–500 μg/kg and the more heavily contaminated samples were destined for pig rations [17].

### 2.2. Comparison of Urinary Mycotoxin/Metabolite Ratios in Naturally Exposed Pigs and Pigs Fed with Experimental Contaminated Diets

**Figure 3:**
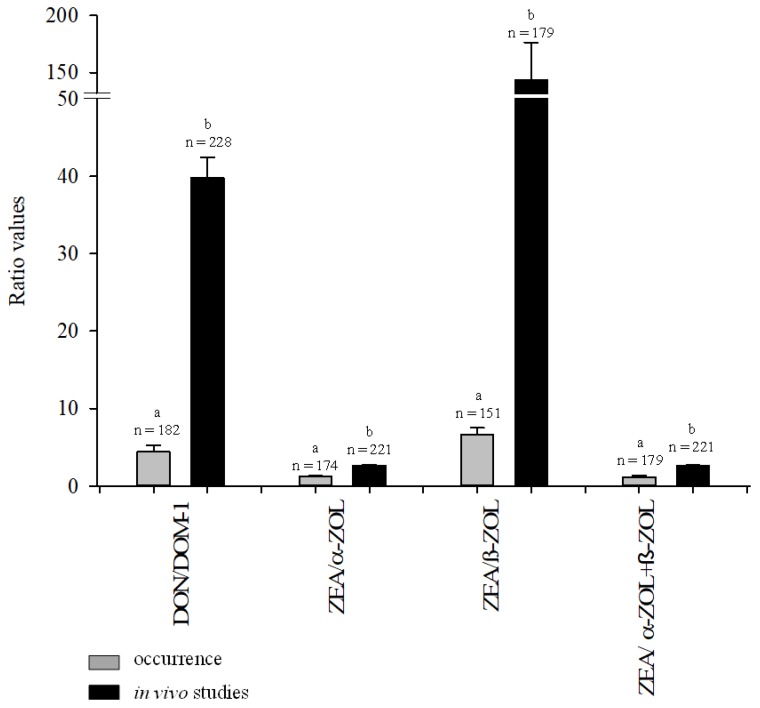
Ratios of DON/DOM-1, ZEA/α-ZOL, ZEA/β-ZOL and ZEA/α-ZOL + β-ZOL in the urine of pigs chronically exposed to naturally contaminated feeds (grey bars) [7,11,13] and pigs exposed for a short period to relatively high doses of DON and/or ZEA (black bars) [6,7,19,20,21,22,23]. ^a,b^ Different letters within each couple of bars represent statistically significant difference (p < 0.05).

In Figure 3, the ratios of DON/DOM-1, ZEA/α-ZOL, ZEA/β-ZOL and ZEA/α-ZOL + β-ZOL measured in the urine of pigs chronically exposed to naturally contaminated feeds are compared to pigs exposed for a short period (1–29 days) to relatively high DON and/or ZEA doses in experimental *in vivo* studies [6,7,18,19,20,21,22]. All mean ratios measured in the occurrence studies [7,11,13], including the present study, were statistically lower (*p* < 0.05) than those measured in *in vivo* studies where pig diets contained higher levels of DON and/or ZEA (Figure 3). The calculated total mean level of DON in feeds consumed by the monitored pigs of the three occurrence studies [7,13], including the present study, was 60 µg/kg, whereas the calculated total mean level of DON in the various feeds used in the seven *in vivo* studies was 2321 µg/kg [6,7,18,19,20,21,22]. The calculated total mean level of ZEA in feeds consumed by pigs of the four occurrence studies [7,11,13], including the present study, was 21 µg/kg, whereas the total mean level of ZEA in various feeds used in the six *in vivo* studies was 920 µg/kg [6,7,18,19,21,23]. The data shown in Figure 3 suggested that pigs chronically exposed to relatively low levels of DON and/or ZEA (occurrence studies) had an improved capacity to metabolize DON into DOM-1 as compared to pigs exposed for a relatively short period to high levels of these mycotoxins, i.e. DON/DOM-1 ratio was lower in occurrence studies as compared to *in vivo* studies. Moreover, the results shown in Figure 3 confirmed that pigs mainly metabolize ZEA into α-ZOL, and to a lesser extent into β-ZOL, i.e. ZEA/α–ZOL ratios were always lower that ZEA/β-ZOL ratios both in occurrence results and *in vivo* results (Figure 3).

## 3. Conclusions

The UPLC-MS/MS method used herein for the determination of urinary biomarkers of DON, FB_1_, OTA, AFB_1_ and ZEA was suitable to measure the low concentrations naturally occurring in pig urine from pigs bred in three Swedish regions. A multiple mycotoxin exposure was detected in all samples and the more frequent mycotoxin combinations were total DON + total ZEA + OTA followed by total DON + FB_1_ + ZEA + OTA. Urinary biomarker concentrations were used to estimate PDI and mycotoxin levels in feeds consumed by pigs. The overall mycotoxin levels in feeds were lower the European limits with the exception of 4% of the samples that were found to be contaminated with levels of either DON, OTA or AFB_1_ that were higher than the recommended/regulatory limits. Within the three regions monitored in this study, the pigs bred in the West region were more exposed to DON and ZEA compared to pigs in the South and East regions. Monitoring of urinary mycotoxin biomarkers was quite effective to assess pig exposure to mycotoxins, the mycotoxin levels in consumed grains and to identify the regions at higher risk for mycotoxin accumulation in the grains produced.

## 4. Materials and Methods

### 4.1. Collection of Samples

The sampling of pig urine, collected from slaughterhouses, was conducted from January–December 2012 by the Swedish National Food Agency. The age of the pigs at slaughter were, on average, 6 months with a range of 5–8 months. The West, South and East regions of Sweden were represented by Västra Götaland county (*n* = 74), Skåne county (*n* = 83) and Kalmar county (*n* = 38), respectively (Figure 2). Soon after collection, urine samples were frozen and sent from the National Food Agency (Uppsala, Sweden) to the Institute of Sciences of Food Production (Bari, Italy) for UPLC-MS/MS analysis of biomarkers of the five agriculturally important mycotoxins, i.e.; DON and DOM-1 for DON, AFM_1_ for AFB_1_, FB_1_ for FB_1_, ZEA, α-ZOL and β-ZOL for ZEA, and OTA for OTA.

### 4.2. Chemicals and Reagents

Standard solutions of mycotoxins and their key metabolites were purchased from Romer Labs Diagnostic (Tulln, Austria). In particular, solutions of DON (100 µg/mL), DOM-1 (50 µg/mL), AFM_1_ (0.5 µg/mL), ZEA (100 µg/mL), α-ZOL (10 µg/mL), β-ZOL (10 µg/mL) and OTA (10 µg/mL) were prepared in acetonitrile (ACN), whereas FB_1_ solution (50 µg/mL) was prepared in acetonitrile–water (50:50 *v/v*). The enzymatic solution β-glucuronidase/sulfatase type H-2 from *Helix pomatia* (specific activity 130,200 units/m L β-glucuronidase, 709 units/mL sulfatase) was purchased by Sigma Aldrich (Milan, Italy). Chromatography-grade methanol (MeOH) and glacial acetic acid were obtained from Carlo Erba (Milan, Italy). Ultrapure water was obtained from a Milli-Q system (Millipore, Bedford, MA, USA). Myco6in1+TM immunoaffinity columns were purchased from Vicam L.P (Watertown, MA, USA). OASIS HLB^®^ columns (60 mg, 3 mL) were purchased from Waters (Milford, MA, USA) and regenerated cellulose filters (0.45 µm) were purchased from Sartorius Stedim Biotech (Goettingen, Germany).

### 4.3. Urine Analysis

The analyses of pig urinary mycotoxin biomarkers (DON, DOM-1, AFM_1_, FB_1_, ZEA, α-ZOL, β-ZOL and OTA) were performed using an ultra-performance liquid chromatography tandem mass spectrometry (UPLC-MS/MS) method reported elsewhere [6,7]. Briefly, 5 mL of pig urine was treated with the aqueous solution of β-glucuronidase/sulfatase type H-2 from *Helix pomatia* to hydrolyze glucuronide- and sulfate- conjugates of mycotoxins and their key metabolites. The digested sample was diluted with water (1:1, *v*/*v*) and purified on a Myco6in1+TM multi-antibody immunoaffinity column and OASIS HLB^®^ column connected in tandem. The purified urine was dried down and reconstituted in 200 µl of mobile phase (MeOH:H_2_O, 20:80, *v*/*v*) and analyzed by UPLC-MS/MS with a triple quadrupole API 5000 mass spectrometer (Applied Biosystems, Foster City, CA, USA), equipped with an ESI interfaced to an Acquity UPLC system comprising a binary pump and a microautosampler (Waters, Milford, MA, USA). Data acquisition and processing was performed with Analyst version 1.5.1 software (Applied Biosystems 2011, Foster City, CA, USA). Detailed chromatographic and mass spectrometric operating conditions are reported elsewhere [24,25]. Crea concentrations in urine samples were analyzed according to an enzymatic method described by [26].

### 4.4. Calibration Curves

Quantification of mycotoxin biomarkers in the 195 purified pig urine sample extracts was performed using matrix-matched calibration curves. For each set of samples (one for each day), matrix-matched calibration solutions were prepared for 6 purified urinary extracts: one aliquot was analyzed as a control and 5 aliquots were used to prepare calibration samples. In particular, aliquots of urine from 5 pigs were pooled and mixed, then 6 aliquots (5 mL each) were purified according to the protocol reported above. After purification, adequate and increasing amounts of standard solutions of DON, DOM-1, AFM_1_, FB_1_, ZEA, α-ZOL, β-ZOL and OTA were added to the 5 purified extracts, dried down, reconstituted in 200 µL of LC–MS/MS mobile phase and filtered. The calibration ranges in the matrix ranged between: 0.1–100 ng/mL for DON, 0.75–24.85 ng/mL for DOM-1, 0.01–7 ng /mL for AFM_1_, 0.10–101.6 ng/mL for FB_1_, 0.03–20.6 ng/mL for β-ZOL and α-ZOL, 0.01–100 ng/mL for ZEA, 0.01–5.01 ng/mL for OTA.

### 4.5. Recovery Experiment

A mixture of 3 blank pig urine samples was used for the recovery experiment of DON, DOM-1, AFM_1_, FB_1_, ZEA, α-ZOL, β-ZOL and OTA. Triplicate experiments were performed. The spiking concentration of each analyte was reported in Table 1. They ranged from 0.9 ng/mL for OTA to 90 ng/mL for DON. Matrix matched calibration curves were used to quantify each analyte. LOD and LOQ were calculated as 3 times and 10 times the noise, respectively.

### 4.6. Censoring

Left-censored analytical results, i.e.; values below the limit of detection (LOD) and the limit of quantification (LOQ), were evaluated with the substitution method as suggested by the European Food Safety Authority (EFSA 2010) [27]. Within the three scenarios proposed by EFSA (lower, middle and upper bound), we used the middle bound approach, i.e.; results below the LOD (or the LOQ), were given the value LOD/2 (or LOQ/2). To calculate the mean concentration in positive samples (Table 3), the values between the LOD and the LOQ were assigned a fixed value of LOQ/2. LOD and LOQ values for all mycotoxins and metabolites were reported in Table 1.

### 4.7. Estimation of Mycotoxin Intake

Calculation of intake estimation was performed for DON, ZEA, FB_1_, OTA and AFB_1_ in pigs using urinary biomarker concentrations measured in this study according Formula (1), reported by [25]:

PDI = (C × V × 100)/(W × E)(1)

where:

PDI: probable daily intake of each mycotoxin (µg/kg body weight);

C: pig urinary biomarker concentration (µg/L);

V: mean 24 h pig urine volume (2.5 L);

W: mean pig body weight (110 kg);

E: mean urinary excretion rate of each mycotoxin in 24 h post dose in piglets (36.8% for total

ZEA, 27.9% for total DON, 2.6% for FB_1_, 2.6% for OTA and 2.5% for AFB_1_ excreted as AFM_1_ [6]).

### 4.8. Estimation of Feed Contamination

The DON, ZEA, FB_1_, OTA and AFB_1_ contamination in feeds consumed by the pigs monitored in this study was calculated using the calculated PDI of each mycotoxin and Formula (2).

ML = PDI × W/V(2)

where:

ML: mycotoxin level in the feed (µg/kg);

PDI: probable daily intake of the mycotoxin (μg/kg body weight);

W: mean pig body weight (110 kg)

V: mean 24 h pig urine volume (2.5 L)

### 4.9. Statistical Analyses of Urinary Biomarker Concentrations and Geographical Areas

Mean, median and standard deviation of the results were calculated using Microsoft Excel 2013 software (Microsoft Corporation, Redmond, WA, USA). Statistical analyses were performed using GraphPad Instat software version 3.00 (Instat 1997, San Diego, CA, USA). Data were subjected to the unpaired t-test (one-tail *p* value). Values were judged to be significantly different if *p* values were < 0.05.

The Mann–Whitney test and the Kruskal–Wallis equality of population rank test were performed in STATA version 12.1 (STATA Corp. 2017, College Station, TX, USA). A *p*-value < 0.05 was considered significant.

## Figures and Tables

**Table 1:** Results of in-house validation of the LC-MS/MS method for mycotoxin biomarkers in pig urine. DON = deoxynivalenol; ZEA = zearalenone; FB_1_ = fumonisin B_1_; OTA = ochratoxin A; DOM-1 = deepoxy-deoxynivalenol; AFM_1_ = aflatoxin M_1_; α-ZOL = α-zearalenol; β-ZOL = β-zearalenol.

Mycotoxin	Spike levels (ng/mL)	Recovery (%)	RSD ^a^ (%)	LOD ^b^ (ng/mL)	LOQ ^c^ (ng/mL)
DON	90	93	6.8	0.18	0.61
DOM-1	45	82	3.6	0.36	1.21
ZEA	45	98	1.1	0.02	0.07
α-ZOL	45	100	0.9	0.04	0.13
β-ZOL	45	96	12.3	0.04	0.15
OTA	0.9	71	5.0	0.006	0.02
FB_1_	18	64	6.7	0.02	0.06
AFM_1_	9.0	96	0.7	0.01	0.03

^a^ RSD: within-day relative standard deviation ^b^ LOD: limit of detection ^c^ LOQ: limit of quantification.

**Table 2:** Urinary concentrations of mycotoxins and their metabolites in samples of Swedish pigs collected in 2012 (*n* = 195).

Mycotoxin ^a^	% Positives ^b^ (n)	Mean ± SD ^c^ (ng/mL)	Median (ng/mL)	Max (ng/mL)	Mean ± SD ^c^ (ng/mg crea)	Median (ng/mg crea)	Max (ng/mg crea)
DON	93% (181)	19.35 ± 49.53	5.91	510.64	17.25 ± 56.28	3.43	491.25
DOM-1	95% (186)	12.89 ± 20.55	4.95	120.63	10.71 ± 27.14	3.75	318.69
Total DON	98% (192)	32.25 ± 59.92	12.46	538.51	27.95 ± 77.64	7.84	809.94
ZEA	92% (179)	2.44 ± 4.39	0.77	28.32	2.32 ± 7.63	0.58	76.68
α-ZOL	90% (176)	2.72 ± 4.78	1.02	33.86	3.31 ± 13.87	0.69	162.47
β-ZOL	81% (158)	0.75 ± 1.59	0.21	14.69	0.73 ± 1.97	0.13	18.15
Total ZEA	99% (194)	5.91 ± 9.78	2.13	65.66	6.37 ± 22.35	1.41	257.29
OTA	95% (185)	0.31 ± 0.65	0.23	7.94	0.39 ± 2.03	0.11	25.57
FB_1_	42% (82)	0.080 ± 0.256	0.01	2.58	0.084 ± 0.256	0.01	2.08
AFM_1_	7% (14)	0.015 ± 0.063	0.01	0.74	0.014 ± 0.057	0.003	0.50

^a^ DON = deoxynivalenol; DOM-1 = deepoxy-deoxynivalenol; Total DON = DON + DOM-1; ZEA = zearalenone; α-ZOL = α-zearalenol; β-ZOL = β-zearalenol; Total ZEA = ZEA + α-ZOL + β-ZOL; OTA = ochratoxin A; FB_1_ = fumonisin B_1_, AFM_1_ = aflatoxin M_1_; crea = creatinine. ^b^ Positives are considered the samples above the LOD, n = number of samples. ^c^ To calculate mean concentrations, values below the LOD were assigned a fixed value of LOD/2, values between the LOD and the LOQ were assigned a fixed value of LOQ/2. SD = standard deviation.

**Table 3:** Occurrence of urinary mycotoxins and their metabolites in pigs from different countries fed with naturally contaminated feeds.

Country	Method	Mean Biomarker Concentrations (ng/mL) in Positive Samples (% Positive Samples)										
		DON	DOM-1	Total DON	OTA	AFM_1_	FB_1_	ZEA	α-ZOL	β-ZOL	Total ZEA	References
Sweden (*n* = 195) ^a^	LC-MS/MS	20.8 (93)	13.5 (95)	32.7 (98)	0.33 (95)	0.15 (7)	0.18 (42)	2.7 (92)	3.0 (90)	0.9 (81)	5.9 (99)	Present study
Croatia (*n* = 11) ^a^	ELISA	na^b^	na	na	na	na	na	40.5 (100)	na	na	na	[10]
Croatia (*n* = 30) ^a^	ELISA	na	na	na	na	na	na	206	na	na	na	[9]
Austria (*n* = 6) ^a^	LC-MS/MS	na	na	na	na	na	na	5.9 (100)	3.2 (100)	2.1 (83)	10.0 (100)	[11]
Belgium (*n* = 19) ^c^	LC-MS/MS	54.6 (74)	na	na	0.11 (26)	nd ^d^	0.30 (21)	nd	nd	nd	nd	[12]
Vietnam (*n* = 15) ^a^	LC-UV/FLD	10.3 (60)	10.3 (40)	na	na	4.1 (80)	na	6.9 (7)	2.8 (47)	10 (7)	19.7 (93)	[13]
Vietnam (*n* = 1920) ^c^	ELISA	na	na	na	na	0.63 (53.9)	na	na	na	na	na	[8]
France (*n* = 28) ^a, e^	LC-MS/MS	12.6 (93)	0.5 (11)	13.4 (11)	0.2 (43)	nd	0.5 (29)	0.5 (89)	0.3 (18)	nd	0.86 (18)	[7]

^a^ using enzymatic hydrolysis prior to analysis. ^b^ not analyzed. ^c^ not using enzymatic hydrolysis prior to analysis. ^d^ not detected. ^e^ piglet urine was collected before the administration of contaminated feed boluses.

**Table 4:** Probable daily intake (PDI) (µg/kg bw^a^) of total DON (DON + DOM-1), total ZEA (ZEA + α-ZOL + β-ZOL), OTA, AFB_1_ and FB_1_ in 195 Swedish pigs.

Analyte	Mean ± SD	Median	Max
Total DON	2.66 ± 4.93	1.03	44.34
Total ZEA	0.36 ± 0.60	0.13	4.06
OTA	0.27 ± 0.57	0.20	6.94
FB_1_	0.07 ± 0.22	0.01	2.26
AFB_1_	0.01 ± 0.06	0.005	0.67

^a^ bw = body weight.

**Table 5:** Comparison of EU limits and mean levels of DON, ZEA, OTA, AFB_1_, and FB_1_ in pig feeds calculated from urinary biomarker concentrations.

Mycotoxin	Limits (µg/kg)	Mean ± SD (µg/kg)	Median (µg/kg)	Max (µg/kg)	*n* > limit East	*n* > limit South	*n* > limit West
DON	900 ^1^	116.84 ± 217.09	45.14	1951.11	0	0	3
ZEA	250 ^1^	16.06 ± 26.57	5.78	178.42	0	0	0
OTA	50 ^1^	11.87 ± 25.02	8.85	305.25	1	0	2
FB_1_	5000 ^1^	3.08 ± 9.84	0.38	99.26	0	0	0
AFB_1_	20 ^2^	0.60 ± 2.51	0.20	29.64	0	1	0

^1^ Commission Recommendation (EC) n. 2006/576. ^2^ Commission Regulation (EC) n. 574/2011.

**Table 6:** Statistical comparison of urinary mycotoxin biomarker concentrations in pigs bred in three Swedish regions.

Mycotoxin	East Region (*n* = 38)			South Region (*n* = 83)			West Region (*n* = 74)		
	Positives (%) ^1^	Mean ± SD (ng/mL)	Mean ± SD (ng/mg crea)	Positives (%) ^1^	Mean ± SD (ng/mL)	Mean ± SD (ng/mg crea)	Positives (%) ^1^	Mean ± SD (ng/mL)	Mean ± SD (ng/mg crea)
DON	90	7.80 ± 12.0^a^	19.8 ± 78.3 ^AB^	94	9.37 ± 11.9 ^a^	6.47 ± 9.29 ^A^	93	36.5 ± 76.3^b^	28.0 ± 70.4 ^B^
DOM-1	90	4.95 ± 4.47 ^a^	6.60 ± 10.3 ^A^	93	9.95 ± 16.2 ^a^	6.41 ± 9.18 ^A^	86	20.3 ± 26.8^b^	17.6 ± 41.6 ^B^
Total DON	100	12.8 ± 13.6 ^a^	26.3 ± 87.5 ^AB^	96	19.3 ± 22.8 ^a^	12.9 ± 15.1 ^B^	99	56.8 ± 88.7^b^	45.7 ± 106 ^A^
ZEA	92	1.93 ± 3.04 ^ab^	3.97 ± 12.8 ^A^	94	1.76 ± 3.89 ^a^	1.33 ± 3.65 ^B^	95	3.47 ± 5.27^b^	2.59 ± 7.39 ^A^
α-ZOL	84	2.62 ± 4.46 ^a^	6.90 ± 26.5 ^A^	96	2.01 ± 5.18 ^a^	1.84 ± 5.97 ^B^	89	3.57 ± 4.38^b^	3.12 ± 10.3 ^A^
β-ZOL	84	1.25 ± 2.72 ^ab^	1.66 ± 3.66^A^	81	0.50 ± 1.07 ^a^	0.51 ± 1.38 ^B^	81	0.77 ± 1.22^b^	0.50 ± 0.89 ^AB^
Total ZEA	100	5.80 ± 8.83 ^ab^	12.5 ± 42.2 ^A^	100	4.27 ± 9.47 ^a^	3.67 ± 10.5 ^B^	99	7.80 ± 10.35^b^	6.21 ± 18.2 ^A^
OTA	85	0.26 ± 0.37 ^a^	0.90 ± 4.13 ^A^	98	0.26 ± 0.16 ^a^	0.19 ± 0.19 ^A^	95	0.39 ± 1.01^a^	0.36 ± 1.43 ^A^
FB_1_	61	0.11 ± 0.41 ^a^	0.11 ± 0.23 ^A^	30	0.09 ± 0.26 ^a^	0.10 ± 0.33 ^A^	50	0.06 ± 0.10^a^	0.05 ± 0.15 ^A^
AFM_1_	0	0.01 ± 0.00 ^a^	0.02 ± 0.08 ^A^	13	0.03 ± 0.09 ^a^	0.02 ± 0.06^A^	5	0.01 ± 0.01^a^	0.01 ± 0.03 ^A^

^1^ % of samples > nd. ^a^ or ^b^ (^A^ or ^B^): the mean concentration, in ng/mL or ng/mg crea, of a specific mycotoxin is not significantly different (*p* > 0.05, Mann–Whitney test) between the three regions if the letter is the same in the same line.
